# *Salix* transect of Europe: additional leaf beetle (Chrysomelidae) records and insights from chrysomelid DNA barcoding

**DOI:** 10.3897/BDJ.7.e46663

**Published:** 2019-11-04

**Authors:** Roy Canty, Enrico Ruzzier, Quentin C Cronk, Diana M Percy

**Affiliations:** 1 Natural History Museum, Cromwell Road, SW7 5BD, London, United Kingdom Natural History Museum, Cromwell Road, SW7 5BD London United Kingdom; 2 Universtità degli Studi di Padova, Legnaro (Padova), Italy Universtità degli Studi di Padova Legnaro (Padova) Italy; 3 Natural History Museum, London, United Kingdom Natural History Museum London United Kingdom; 4 University of British Columbia, Vancouver, Canada University of British Columbia Vancouver Canada

**Keywords:** Salicophagy, salicivorous insects, Salicaceae, Chrysomelidae, DNA barcoding, Europe, megatransect

## Abstract

Occurrence patterns of chrysomelid beetles (Coleoptera: Chrysomelidae), associated with willow (*Salix* spp.) at 42 sites across Europe, have previously been described. The sites form a transect from Greece (lat. 38.8 °N) to arctic Norway (lat. 69.7 °N). This paper reports additional records and the results of DNA sequencing in certain genera. Examination of further collections from the transect has added 13 species in the genera *Aphthona, Chrysomela, Cryptocephalus, Epitrix*, *Galerucella* (2 spp.), *Gonioctena, Phyllotreta* (2 spp.), *Pachybrachis* (3 spp.) and *Syneta.* We also report the sequencing of the DNA regions cytochrome oxidase 1 (CO1) and cytochrome B (cytB) for a number of samples in the genera *Plagiodera, Chrysomela, Gonioctena, Phratora, Galerucella* and *Crepidodera.* The cytB sequences are the first available for some of these taxa. The DNA barcoding largely confirmed previous identifications but allowed a small number of re-assignments between related species. Most notably, however, it was evident that the southernmost material (Greece and Bulgaria) of specimens, previously treated as *Crepidodera aurata* sens. lat., belonged to a distinctive molecular cluster. Morphological re-examination revealed these to be *C. nigricoxis* Allard, 1878. This is an example of how morphotaxonomy and DNA barcoding can work iteratively to refine identification. Our sequences for *C. nigricoxis* appear to be the first available for this taxon. Finally, there is little geographic structure evident, even in widely dispersed species.

## Introduction

Since early pleas were made for the routine incorporation of a molecular component to taxonomy (“DNA barcoding”) (Hebert et al. 2003a, Hebert et al. 2003b, Tautz et al. 2003), a large amount of literature has accrued and a very large number of sequences backed by voucher specimens have been deposited in standard databases. It is now well established that, in many animal groups, sequencing mitochondrial cytochrome c oxidase subunit 1 (COI) provides a straightforward way of gaining taxonomic insight. Early concerns about molecular methods being somehow antagonistic to morphological taxonomy have given way to acceptance that molecular and morphological taxonomy are complementary, reciprocally illuminating and iterative processes.

As part of a study of lowland willow communities sampled from south to north across Europe, we have previously investigated the occurrence and abundance patterns of chrysomelid beetles (Coleoptera: Chrysomelidae) associated with *Salix* species (Canty et al. 2016). In this study, large numbers of individual beetles were processed and it was impossible with available resources to perform large numbers of genitalia dissections. For this reason, a broad morphospecies concept was used, identifying to species largely using external morphology. We have now been able to test some of these morphospecies assignments using DNA barcoding. This paper reports the new insights that this offers. We also take the opportunity to report additional chrysomelid records from the transect following examination of additional collections.

## Material and methods

### Collecting methods

Chrysomelid beetles were collected from willows (*Salix* spp.) by the authors ER and DP at all sites, as previously described (Canty et al. 2016). Details of the sites and the method of their selection have been given in previous papers (Cronk et al. 2015; Canty et al. 2016). The sample sites formed a megatransect from Greece to arctic Norway (Table 1). All collections are deposited in the Natural History Museum, London (BMNH).

### Specimen examination and analysis

Morphological procedures followed those used in Canty et al. (2016). A selected subset of specimens was chosen for sequencing (Table 2). These included specimens deemed to be potentially problematic in the original identifications and samples from widespread and variable species. DNA was extracted from material preserved in 90% ethanol. Sequences of mitochondrial cytochrome oxidase subunit 1 (COI) and cytochrome B (cytB) were obtained following protocols for DNA extraction, polymerase chain reaction (PCR) and sequencing described in Percy et al. (2018) with additional primers used for COI (LCO1490 and HCO2198; Folmer et al. 1994). As numerous COI sequences are available on GenBank, we were able to align our own sequences with previously published ones (Table 3). Aligned sequences were analysed using neighbour-joining (NJ) with uncorrected (p) distances in PAUP* (Swofford 2003). Bootstrap support was obtained using 1000 replicates. Sequences generated as a result of this study are all deposited in GenBank (accession numbers MN629748 - MN629886) (Table 2).

## Results

### Taxonomic insights from molecular barcoding

**Figure 1.**
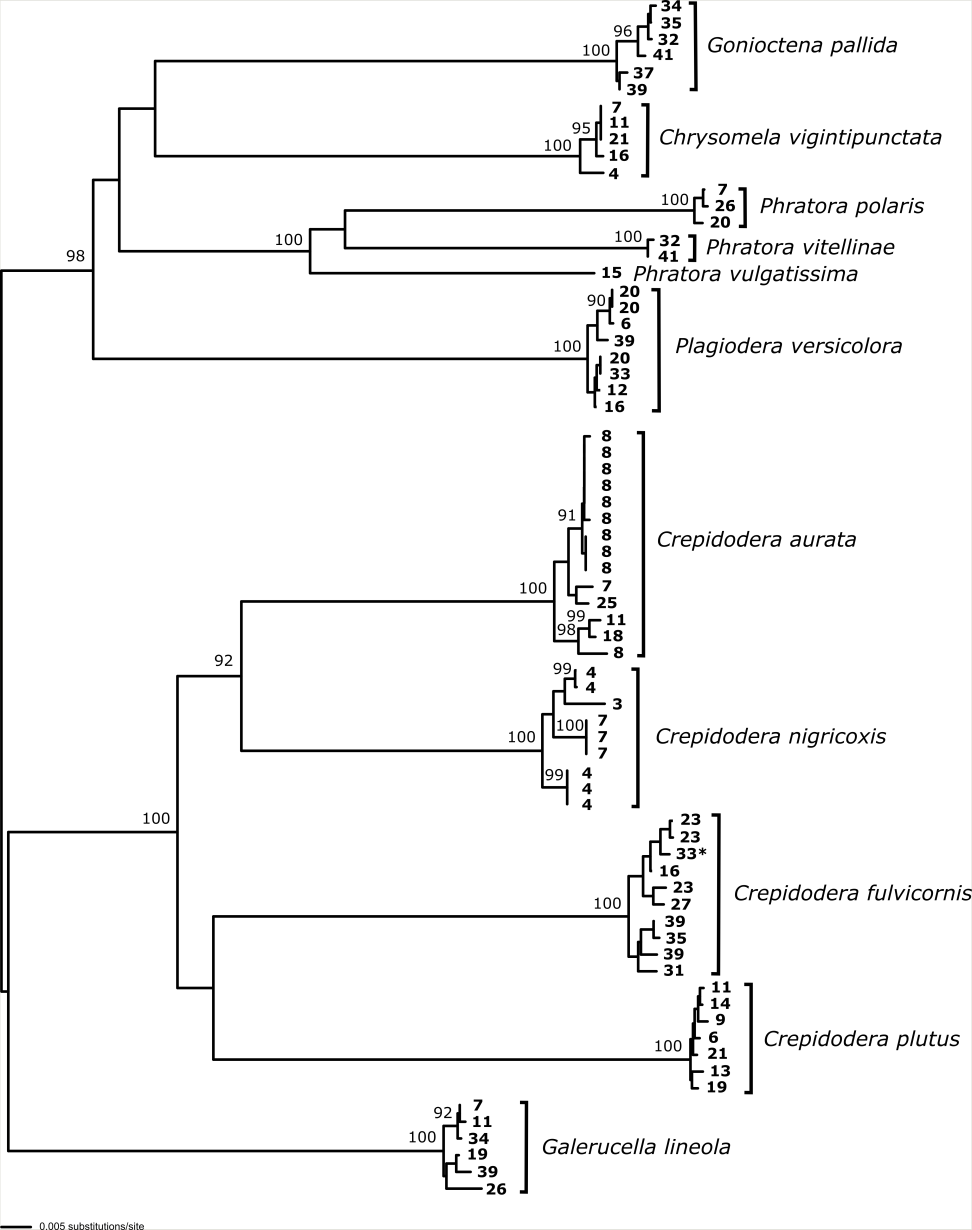
DNA analysis (NJ tree) using COI and cytB sequences generated in this study. Node support shown only for nodes ≥ 90% bootstrap support.

**Figure 2.**
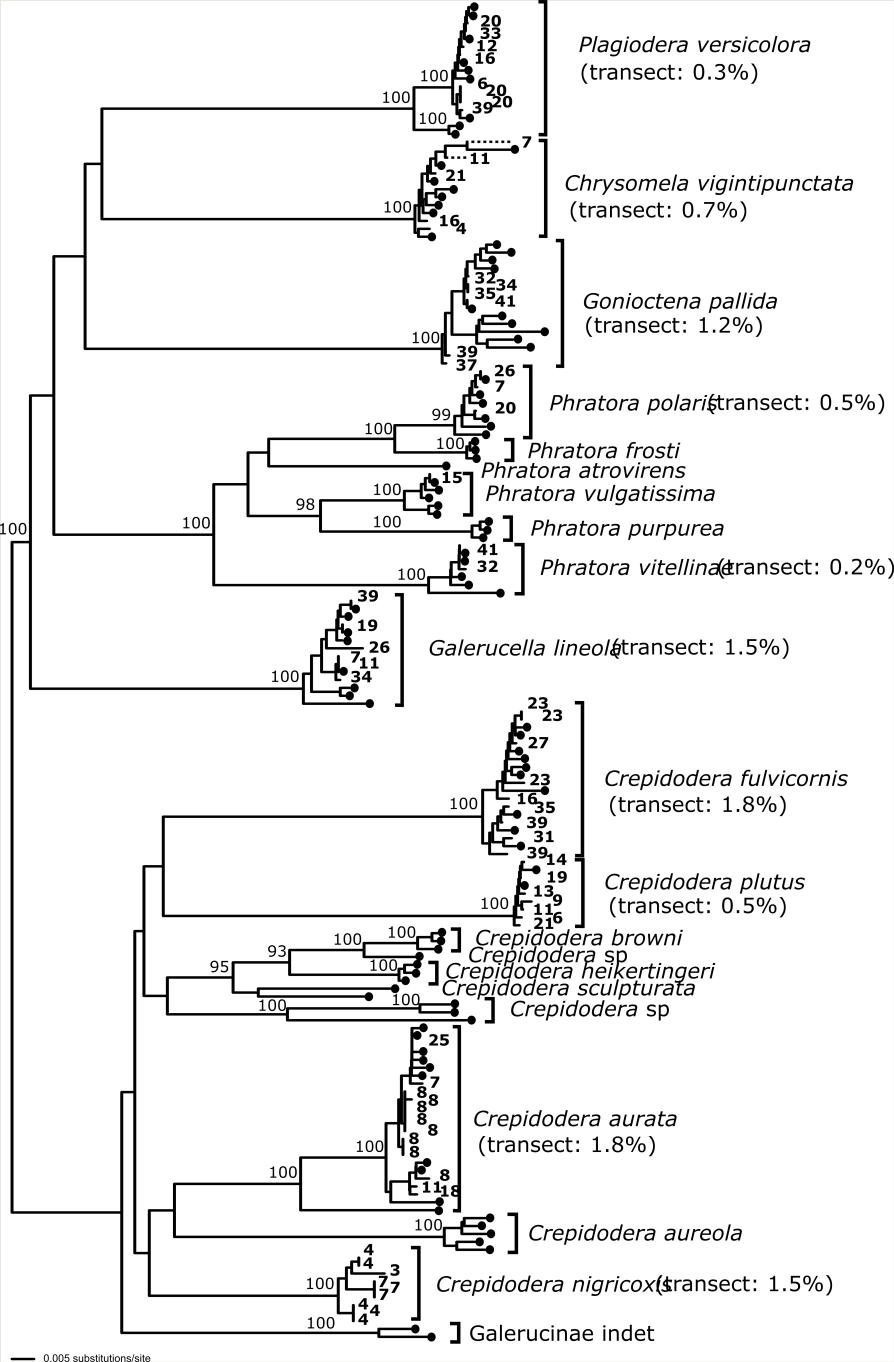
DNA barcoding analysis using COI sequences generated in this study and from GenBank. Sequences from this study show the site number and those obtained from GenBank are indicated by a black circle (GenBank accessions given in Table 3). Node support shown only for nodes > 90% bootstrap support. Maximum intraspecific divergences are shown (for our transect samples only), estimated using uncorrected (p) distances (see methods).

We used DNA sequencing to test and, if necessary, refine our morphospecies assignments made previously (Canty et al. 2016). Generally, the barcoding results confirmed the morphospecies assignments and provide well-supported species clusters (Figs 1, 2). However, the Chrysomelidae barcoding analysis revealed that some specimens were incorrectly assigned in Canty et al. (2016) (Table 2; Fig. 2). These were all due to using broad morphospecies concepts for *Phratora vitellinae* (Linnaeus, 1758) and *Crepidodera aurata* Marsham, 1802. In *Phratora*, three specimens assigned to *Phratora vitellinae* clustered in the barcoding data with sequences identified on GenBank as *P. polaris* Schneider, 1886; and one specimen assigned to *Phratora vitellinae* clustered with GenBank sequences of *P. vulgatissima* (Linnaeus, 1758). In *Crepidodera*, two specimens assigned to *Crepidodera aurata* clustered with GenBank sequences, plus our own sequences, for *C. fulvicornis* Fabricius, 1792.

**Figure 3.**
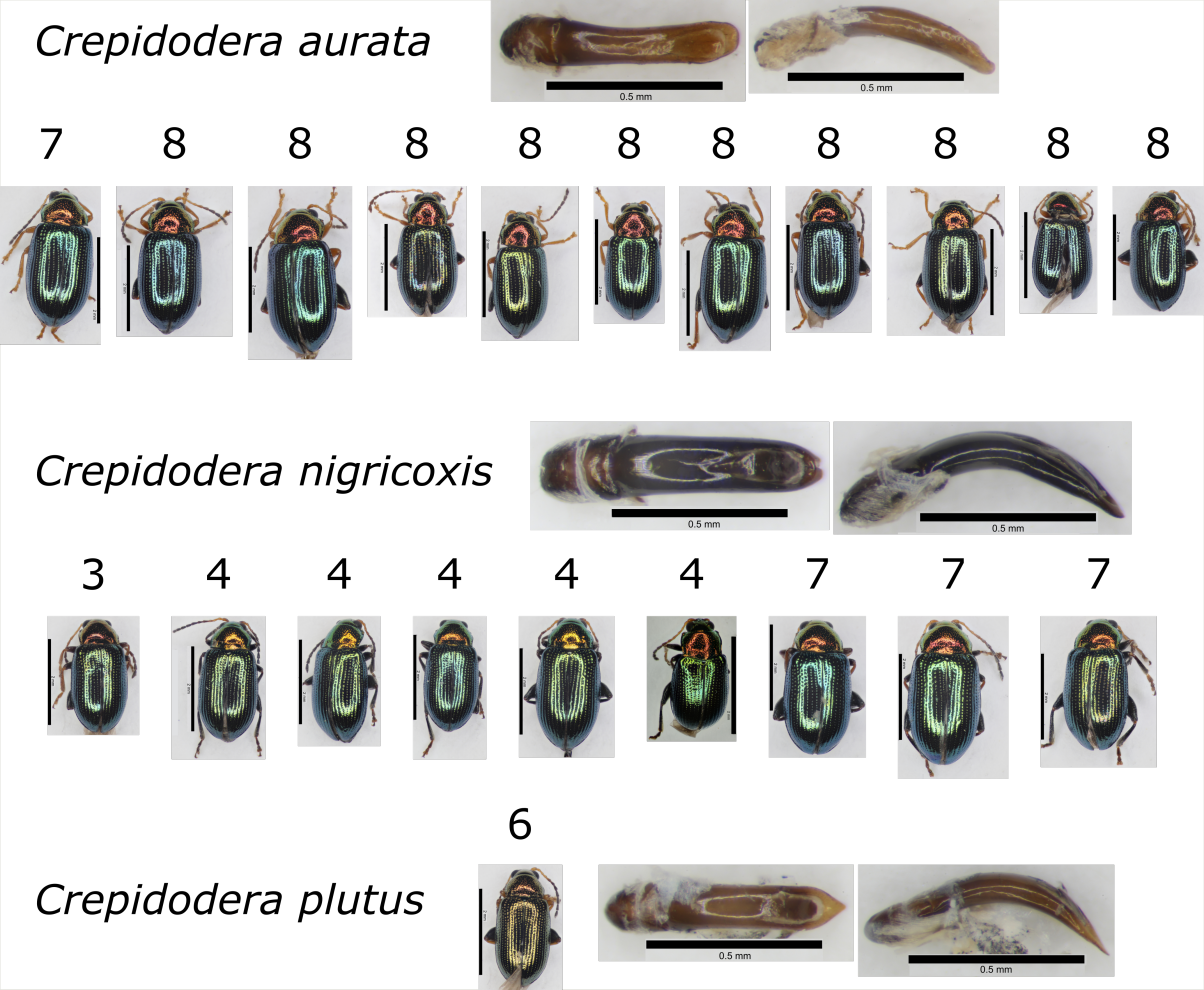
Comparative figure of similar species in the genus *Crepidodera* Dejean, 1836 species, showing size and colour variation of *Crepidodera aurata* Marsham, 1802 and *C. nigricoxis* Allard, 1878, with an example of *Crepidodera plutus* (Latreille, 1804) for comparison. Site number given for each individual. Scale bars whole insect = 2 mm, aedeagus = 0.5 mm. DNA barcoding clearly distinguishes the species.

In addition, we noted that certain specimens assigned to *Crepidodera aurata* formed a distinct molecular cluster, distinct from our own *C. aurata* sequences and from all others downloaded from GenBank. These specimens were the southernmost specimens of our *C. aurata* from sites 3 and 4 (Greece) and site 7 (Bulgaria). This prompted a morphological re-examination of these samples, including dissections of genitalia and these specimens were identified with *C. nigricoxis* Allard, 1878 (Fig. 3; Table 2). The two species are very similar in external morphology and variable (Fig. 3). Nevertheless, the molecular data clearly separates them (Figs 1, 2). Our sequences for *C. nigricoxis* appear to be the first to be made available for this taxon. Gavrilović and Ćurčić (2013) note that *C. nigricoxis* is found on *Salix alba* L. Although we did not distinguish willow species at the point of collection, *Salix alba* was present at all the sites where we recorded *C. nigricoxis* (Cronk et al. 2015).

Finally, our analysis indicates that a specimen from GenBank (KM442534.1: voucher GBOL_Col_FK_7108), identified as *Phratora tibialis* (Suffrian, 1851), may in fact be *P. polaris* (Table 3; Fig. 2).

### Phylogeographic patterns

There is little phylogeographic structure evident from the sequence data, even for widely dispersed taxa along the transect. Fig. 2 (COI data) is suggestive of a split in *Crepidodera fulvicornis* between northern samples (Finland: 31, 35, 39) in one clade and southern samples (Hungary: 16, Poland: 23, Latvia: 27) in the other (e.g. a zoogeographic boundary around Estonia or the Gulf of Finland), but one sample from Finland (site 33) that only sequenced for cytB (Fig. 1) clusters with the southern clade. The absence of clear phylogeographic patterns in the chrysomelids is similar to our findings for curculionids (Canty et al. in review), but differs from those found in a hemipteran taxon (the nettle psyllid; Psylloidea, Hemiptera) sampled along the transect in which population structure suggests distinct regional clades (Wonglersak et al. 2017).

### Additional chrysomelid records from the transect

Since the publication of Canty et al. (2016), examination of additional material from general collections by DP over the transect has brought to light some further records (all single individuals per site, unless otherwise stated). The additional records are: *Aphthona cf. lutescens* (Gyllenhal, 1808) (site 22); *Chrysomela lapponica* Linnaeus, 1758 (site 40 and also in supplementary site ii-I [site details in Cronk et al. 2015]); *Cryptocephalus ocellatus* Drapiez, 1819 (site 20a); *Epitrix* sp. (site 22 - two individuals); *Galerucella cf. nymphaeae* (Linnaeus, 1758) (site 37); *Galerucella cf. sagittariae* (Gyllenhal, 1813) (site 38); *Gonioctena cf. olivacea* (Forster, 1771) (site 39); *Phyllotreta cf. vittula* (Redtenbacher, 1849) (site 24); *Phyllotreta undulata* (Kutschera, 1860) (sites 27, 30); *Pachybrachis hieroglyphicus* Laicharting, 1781 (site 20a); *Pachybrachis* sp. (site 20); *Pachybrachis cf. salfii* Burlini, 1956 (site 31) ; and *Syneta* sp. (site 35). Some of these are not generally associated with willows and are probably accidental by-catch (e.g. *Galerucella nymphaeae* and *Galerucella sagittariae*). These additional records do not materially change the basic data or conclusions of Canty et al. (2016), but bring the total number of species to 47 (not 34).

## Discussion

The barcoding, described here, provides a good example of the value of iterative molecular and morphological processes in taxonomy. In this case, a broad morphospecies concept allowed determination of those species that have the greatest geographic and morphological variation. These could then be targeted for barcoding to determine patterns of molecular variation. In the case of *Crepidodera aurata* sens. lat., this led to the distinguishing of two divergent molecular clusters. This in turn led to a re-appraisal of the morphology and to the refinement of the concept of *C. aurata* and the recognition of *C. nigricoxis* as its apparent replacement (at least in our sampling) in southern Europe (Greece and Balkans). This very small example thus serves to emphasise that morphological and molecular taxonomy, taken together and applied iteratively, are powerful adjuncts.

## Figures and Tables

**Table 1.** Basic site details. See Cronk et al. (2015) for further details.

SITE#	Country	Lat N	Long E	Alt (m)	Date of collection
1	Greece	38.80007	22.4629	37	21-iv-2015
2	Greece	38.902	22.31015	33	21-iv-2015
3	Greece	39.306694	22.528323	177	22-iv-2015
4	Greece	40.032685	22.175437	534	22-iv-2015
5	Greece	41.113317	23.273893	31	23-iv-2015
6	Bulgaria	41.412468	23.318609	90	23-iv-2015
7	Bulgaria	42.165622	22.998141	392	24-iv-2015
8	Bulgaria	42.923989	23.810563	339	24-iv-2015
9	Bulgaria	43.739343	23.966755	35	24-iv-2015
10	Romania	44.260343	23.786781	81	25-iv-2015
11	Romania	44.961981	23.190337	172	25-iv-2015
12	Romania	45.510676	22.737225	556	26-iv-2015
13	Romania	46.518504	21.512839	102	26-iv-2015
14	Hungary	46.700744	21.31268	94	27-iv-2015
15	Hungary	47.665648	21.261768	91	27-iv-2015
16	Hungary	48.374291	20.725264	148	28-iv-2015
17	Poland	49.463447	21.697255	385	28-iv-2015
18	Poland	50.470234	22.238372	157	29-iv-2015
19	Poland	50.673994	21.823391	141	29-iv-2015
20	Poland	51.775039	21.1971	101	30-iv-2015
20a	Poland	51.775039	21.1971	101	11-vi-2015
21	Poland	52.69398	21.8529	96	12-vi-2015
22	Poland	53.55483	22.30299	128	12-vi-2015
23	Poland	54.06943	23.11745	137	13-vi-2015
24	Lithuania	54.92583	23.7742	28	13-vi-2015
25	Lithuania	55.79557	24.56678	62	13-vi-2015
26	Latvia	56.71141	24.25162	23	14-vi-2015
27	Latvia	57.74963	24.4023	7	14-vi-2015
28	Estonia	58.42257	24.44063	18	15-vi-2015
29	Estonia	59.40289	24.93577	48	15-vi-2015
30	Finland	60.27299	24.65843	33	16-vi-2015
31	Finland	61.09965	25.6282	84	16-vi-2015
32	Finland	62.04962	26.12369	174	17-vi-2015
33	Finland	63.01589	25.80457	139	17-vi-2015
34	Finland	64.05074	25.52664	91	17-vi-2015
35	Finland	64.61287	25.53805	58	18-vi-2015
36	Finland	65.32835	25.29175	1	18-vi-2015
37	Finland	66.24947	23.8945	51	19-vi-2015
38	Finland	67.21253	24.12629	160	19-vi-2015
39	Finland	67.91183	23.63411	233	19-vi-2015
40	Norway	68.8138	23.26658	374	20-vi-2015
41	Norway	69.72487	23.40581	289	20-vi-2015
42	Norway	70.65234	23.66583	67	21-vi-2015

**Table 2.** Samples sequenced in this study, reassignments made, and sequences deposited in GenBank: COI (cytochrome oxidase 1), cytB (cytochrome B).

**Original species ID**	**Reassignment ID**	**Site**	**COI**	**cytB**
*Chrysomela vigintipunctata*	correct	4	MN629768	MN629838
*Chrysomela vigintipunctata*	correct	7	MN629769	MN629839
*Chrysomela vigintipunctata*	correct	11	MN629770	MN629840
*Chrysomela vigintipunctata*	correct	16	MN629771	MN6298341
*Chrysomela vigintipunctata*	correct	21	MN629772	MN629842
*Crepidodera aurata*	*Crepidodera nigricoxis*	3	MN629760	MN629830
*Crepidodera aurata*	*Crepidodera nigricoxis*	4	MN629762	MN629832
*Crepidodera aurata*	*Crepidodera nigricoxis*	4	MN629763	MN629833
*Crepidodera aurata*	*Crepidodera nigricoxis*	4	MN629764	MN629834
*Crepidodera aurata*	*Crepidodera nigricoxis*	4	MN629765	MN629835
*Crepidodera aurata*	*Crepidodera nigricoxis*	4	MN629773	MN629843
*Crepidodera aurata*	*Crepidodera nigricoxis*	7	MN629761	MN629831
*Crepidodera aurata*	*Crepidodera nigricoxis*	7	MN629766	MN629836
*Crepidodera aurata*	*Crepidodera nigricoxis*	7	MN629767	MN629837
*Crepidodera aurata*	correct	7	MN629759	MN629829
*Crepidodera aurata*	correct	8	MN629749	MN629819
*Crepidodera aurata*	correct	8	MN629750	MN629820
*Crepidodera aurata*	correct	8	MN629751	MN629821
*Crepidodera aurata*	correct	8	MN629752	MN629822
*Crepidodera aurata*	correct	8	MN629753	MN629823
*Crepidodera aurata*	correct	8	MN629754	MN629824
*Crepidodera aurata*	correct	8	MN629755	MN629825
*Crepidodera aurata*	correct	8	MN629756	MN629826
*Crepidodera aurata*	correct	8	MN629757	MN629827
*Crepidodera aurata*	correct	8	MN629758	MN629828
*Crepidodera aurata*	correct	11	MN629774	MN629844
*Crepidodera aurata*	correct	18	MN629775	MN629845
*Crepidodera aurata*	correct	25	MN629776	MN629846
*Crepidodera aurata*	*Crepidodera fulvicornis*	33	/	MN629847
*Crepidodera aurata*	*Crepidodera fulvicornis*	39	MN629777	MN629848
*Crepidodera fulvicornis*	correct	16	MN629778	/
*Crepidodera fulvicornis* (a)	correct	23	MN629779	/
*Crepidodera fulvicornis* (b)	correct	23	MN629780	MN629849
*Crepidodera fulvicornis* (c)	correct	23	MN629781	MN629850
*Crepidodera fulvicornis*	correct	27	MN629782	MN629851
*Crepidodera fulvicornis*	correct	31	MN629783	MN629852
*Crepidodera fulvicornis*	correct	35	MN629784	MN629853
*Crepidodera fulvicornis*	correct	39	MN629785	MN629854
*Crepidodera plutus*	correct	6	MN629748	MN629818
*Crepidodera plutus*	correct	9	MN629786	MN629855
*Crepidodera plutus*	correct	11	MN629787	MN629856
*Crepidodera plutus*	correct	13	MN629788	MN629857
*Crepidodera plutus*	correct	14	MN629789	MN629858
*Crepidodera plutus*	correct	19	MN629790	MN629859
*Crepidodera plutus*	correct	21	MN629791	MN629860
*Galerucella lineola*	correct	7	MN629792	MN629861
*Galerucella lineola*	correct	11	MN629793	MN629862
*Galerucella lineola*	correct	19	MN629794	MN629863
*Galerucella lineola*	correct	26	MN629795	MN629864
*Galerucella lineola*	correct	34	MN629796	MN629865
*Galerucella lineola*	correct	39	MN629797	MN629866
*Gonioctena pallida*	correct	32	MN629798	MN629867
*Gonioctena pallida*	correct	34	MN629799	MN629868
*Gonioctena pallida*	correct	35	MN629800	MN629869
*Gonioctena pallida*	correct	37	MN629801	MN629870
*Gonioctena pallida*	correct	39	MN629802	MN629871
*Gonioctena pallida*	correct	41	MN629803	MN629872
*Phratora vitellinae*	*Phratora polaris*	7	MN629804	MN629873
*Phratora vitellinae*	*Phratora vulgatissima*	15	MN629805	MN629874
*Phratora vitellinae*	*Phratora polaris*	20	MN629806	MN629875
*Phratora vitellinae*	*Phratora polaris*	26	MN629807	MN629876
*Phratora vitellinae*	correct	32	MN629808	MN629877
*Phratora vitellinae*	correct	41	MN629809	MN629878
*Plagiodera versicolora*	correct	6	MN629810	MN629879
*Plagiodera versicolora*	correct	12	MN629811	MN629880
*Plagiodera versicolora*	correct	16	MN629812	MN629881
*Plagiodera versicolora* (a)	correct	20	MN629813	MN629882
*Plagiodera versicolora* (b)	correct	20	MN629814	MN629883
*Plagiodera versicolora* (c)	correct	20	MN629815	MN629884
*Plagiodera versicolora*	correct	33	MN629816	MN629885
*Plagiodera versicolora*	correct	39	MN629817	MN629886

**Table 3.** GenBank sequences included in the phylogenetic analysis. The sample in **bold** under *Phratora polaris* was downloaded from GenBank as *P. tibialis*.

**Species (Chrysomelidae)**	**GenBank Accession numbers**
*Chrysomela vigintipunctata*	AY027624, KM451318, KM443123, JN087422, KU188452, KM443640, KJ961764, KM443492
*Crepidodera aurata*	KJ966066, KJ962544, KF654801, KF656415, KF654798, KJ963892, KM450642, KM445873, KM448484, KM445803
*Crepidodera aureola*	KF655591, KF655792, KF655954, KF652694, KF652646
*Crepidodera browni*	KR487413, KR481606, KR490696
*Crepidodera fulvicornis*	KF656356, KM448864, KF656033, KF656133, KF656534, KF656533, KF655283, KJ963238, KJ964506, KJ962307
*Crepidodera heikertingeri*	KR487651, KT608408, KT608832
*Crepidodera plutus*	KM452345, KM441553
*Crepidodera sculpturata*	KR486405
*Crepidodera* sp.	KM849066, KR490063, KR483107, KR483276, KM845706
*Galerucella lineola*	KJ963510, KF652931, KC336454, KJ966162, KC336452, KF652986, KF652930, KM439994
Galerucinae sp.	KR485283, KR487847
*Gonioctena pallida*	FJ346952, FJ346941, FJ346950, FJ346944, KJ962854, FJ346935, FJ346934, FJ346975, FJ346931, FJ346859
*Phratora atrovirens*	KJ965539
*Phratora frosti*	KM841607, KM846081, KR119812
*Phratora polaris*	KJ965979, KM449319, KJ963698, **KM442534**, KM848244, KJ967261
*Phratora purpurea*	KM845219, KR481952, KM845523
*Phratora vitellinae*	KM443624, KJ963556, KJ963944, KM447598, KF656305
*Phratora vulgatissima*	KJ962797, KF656615, KF656399, KM445038, KM442140
*Plagiodera versicolora*	KR480773, KR483766, KM439446, KJ962066, KF656648, KF652968, KF652966, KF656252, KF656237
